# Foggy connections, cloudy frontiers: On the (non-)adaptation of lexical structures

**DOI:** 10.3389/fpsyg.2023.1115832

**Published:** 2023-03-01

**Authors:** Matthias Urban

**Affiliations:** Center for Advanced Studies “Words, Bones, Genes, Tools”, University of Tübingen, Tübingen, Germany

**Keywords:** colexification, language, environment, Central Andes, Quechua

## Abstract

While research on possible adaptive processes in language history has recently centered mostly on phonological variables, here, I return the focus on the lexicon in two different ways. First, I take up the familiar theme of the responsiveness of language structure to the local conditions at different elevations of the earth’s surface by exploring further the idea that language communities at high altitudes may tend not to distinguish lexically, as, e.g., English does, between “cloud” and “fog.” Analyses of a global dataset of languages as well as in-depth study of the languages of the Central Andes are consistent in showing a wide spread of colexification of “cloud” and “fog” across elevations, whereas distinguishing languages tend more to be spoken at lower elevations. Statistically, there is global support for the idea that colexification is triggered by high elevation, but a closer look, in particular at the Andean dataset, paints a more nuanced picture. Concretely, it shows that in some language families, there are consistent preferences for either colexifying or distinguishing between “cloud” and “fog.” In particular, the behavior of the large Quechuan family, which ranges across high- and low-elevation environments but still is consistently colexifying, shows no evidence for adaptive processes within language families. This result is open to various interpretations and explanations, for they suggest lineage-specific preferences for or against colexification that run counter to global trends. It is also at odds with the notions of “efficient communication” and “communicative need” as far as they relate to lexical categories and bars mechanistic or deterministic views on the processes in which the categories of languages are molded.

## 1. Introduction

When linguist Donald Laycock was roaming the highlands of New Guinea in the 1960s to survey and document basic vocabulary in New Guinea languages, he noted several issues in the New Guinea context with the so-called Swadesh list that is often used for that purpose. One of these was that, especially in highland languages, two meanings of the Swadesh list, “cloud” and “fog,” often were expressed by the same form (Laycock, 1970: 1138), or “colexified” as the phenomenon is now commonly called in cross-linguistic studies. While Laycock remained implicit about the underlying reason for the phenomenon—his accompanying notes to the primary data are short and concise—, it seems obvious from his remarks that he considered it to be related to differences in elevation.

From a physical perspective, there is no significant difference between cloud and fog, phenomena which so many languages of the world distinguish lexically: Both are aerosols that consist of tiny water droplets suspended in the earth’s atmosphere, fog at levels close to the ground and clouds higher up. This is also reflected in the lexicon of some languages (Urban, 2012: 470); to stay in New Guinea, in the Kyaka language, for instance, “fog” is *yuu kupa*, literally “low cloud” (Draper and Draper, 2002).

What seems to be underlying Laycock’s comment is the observation that at high elevations, there may be no strong stimulus to distinguish between “cloud” and “fog” lexically as clouds form so close to the points from where they are observed by humans that the essential identity of the phenomena becomes obvious to language users. Regier et al. (2016), who provide a theoretical framework to account for such phenomena, would say that there is no “communicative need” to distinguish “cloud” and “fog” in languages spoken in regions like the New Guinea highlands. Hence, category systems evolve that do not encode an altitude difference in the domain of atmospheric aerosols (“cloud”: water aerosol at high altitude, “fog”: water aerosol at low altitude; though see further below for some qualifications).

The following piece of verbal art from the Central Andes, redacted in the Quechua language as spoken in Southern Peru and taken from Montoya et al.’s (1987: 127) anthology, might reflect the typical ambiguity of terms for “cloud” and “fog” in languages that colexify the two phenomena. In Quechua, the relevant item is *puyu*:

*Chimpa urqupis*

*puyu tiyachkan.*

*Manas puyuchu*

*chayllay puyuqa.*

*Warma yanaypa*

*llantuchallansi*

*puyu tukuspa*

*llantullawachkan.*

“Across there on that mountain is a *puyu*. It is not a *puyu*, just that *puyu*. They say it’s the shadow of my lover which, pretending to be *puyu*, enshrouds me.”

In this poem, *puyu* has the characteristic individuatability of certain types of clouds (e.g., cumulus)—one can speak of a partticular *puyu* on the mountain. But at the same time, there also is the enshrouding quality of fog that is explicitly referenced in the comparison to the lyrical ego’s lover.

The case of “cloud” and “fog” is similar and different in several ways from the one of “snow” and “ice” which Regier et al. (2016) studied. Similar to ice, and infamously snow (Martin, 1986), clouds come in kinds. Fog resembles stratus clouds, but usually not so much the typically stripe-shaped cirrus clouds that form high up in the atmosphere, nor the perceptually clearly individuated cumulus clouds. While these differences might well affect lexicalization and, in particular, colexification patterns, like Regier et al. (2016), I will abstract away from the differentiation between different types of “cloud” and “fog” that languages may or may not make in the empirical parts of this article. But the case is also different in ways that might be relevant to how lexical category systems are shaped by how language users relate to and engage with their environment. Regier et al. (2016) argued that the “local physical environment … shapes local cultural communicative needs” and the category system that evolves in languages does so to cater to these needs efficiently. What seems to be at stake in the case of “cloud” and “fog”, however, is that a perceptual difference between configurations of aerosols in the atmosphere that English speakers are used to calling *cloud* and *fog* is arguably reduced or even does not manifest itself at all in certain environments. In other words, in these environments, there is no “local cultural communicative need” to distinguish the two because, in the most extreme case, they simply may not be distinguishable. This is a slight difference from the framework assumed by Regier, in which, rather, the prevalence of the natural phenomena in question is highlighted. The predicted outcome, however, would be the same: The local physical environments trigger differences in category systems that are lexically reflected in the languages of the world.

By hypothesis, many of the relevant environments would be high-altitude environments. In exploring whether this prediction is borne out, however, we must reckon with considerable differences in the precise orographic conditions of these environments. These differences can affect precipitation, atmospheric moisture levels, etc. in very different ways on micro- and meso-scales. Therefore, the effect of altitude on lexical structure may be non-stationary and/or not significant in some high-altitude environments at all.

Spurred by the first-hand observation by Laycock (1970), in Urban (2012), I have looked at a small sample of 78 languages of the world and recorded whether there are distinct terms for “cloud” and “fog” in dictionaries and/or other lexical sources or a single general term that is translatable as either. There is a third way in which languages may treat “cloud” and “fog” linguistically, which has been distinguished as a separate category in this study: In some languages, like Kyaka, there are morphologically complex terms for “fog” whose head is the word for “cloud” and which is accompanied by different modifiers. The sample is genealogically stratified, i.e., it samples only one language per language family, thereby avoiding phylogenetic dependencies. Results were suggestive: Even though, as I cautioned earlier, sheer elevation may be too coarse a measure of the relevant environmental properties and more nuanced modeling of the local geophysical environment may alter the results and their interpretation, on the basis of elevation data from the GTOPO30 digital elevation model, I was able to report a Spearman’s correlation of ρ∼0.38 that was significant at *p* < 0.001.

I do not think that the case can be settled on the basis of this simple analysis, however the variability of orographic and precipitation conditions in high-elevation regions that might render sheer elevation too simple a variable to test the hypothesized connections in a fine-grained manner aside, there are several concerns that I address here.

One concern mentioned in the original study is its insensitiveness to synonyms: Languages were counted as being of the colexifying kind if there was a general term that sources indicated as covering both “cloud” and “fog,” regardless of whether there were additional, more specific terms, that only denote one of the two. This is a coding decision that may obscure innovations that are precisely of interest in an adaptation-based framework, such as the introduction, *via* borrowing or word-formation, of new terms that are not colexifying, or semantic change in existing terms to cover a gap in the lexicon.

A second concern is analytic, especially in light of the relevant post-2012 literature that achieved a degree of considerable analytic and methodological sophistication in exploring possible effects at the interface of language and environment, including multi-angle explorations of the same suspected relationships (Roberts, 2018). This is something that the Urban (2012) study fell short of.

A third concern, especially in light of Regier et al. (2016), is the wide spread of colexifying languages regarding elevation in the original study. While, as expected, differentiating languages clustered notably in low-altitude regions, colexifying languages occurred at both low and very high elevations. This pattern is the opposite of that observed by Regier et al. (2016): In their study, it was the colexifying languages that were more strongly constrained with regard to the non-linguistic predictor variable, the temperature in their case, whereas the differentiating languages occurred in all climates. This pattern they attributed to “reduced pressure for precise communication about ice and snow in warm climates, and greater pressure for such communication in cold climates.” For “cloud” and “fog,” then, we would have to surmise that there is some sort of “incentive” in low-lying environments to distinguish the two, but freedom to do so or not otherwise, especially in those regions where perceptual boundaries would be blurred to the extent that the distinction between cloud and fog made in languages like English lose their meaningfulness, such as the New Guinea highlands. This seems counterintuitive at least when following Regier et al.’s (2016) logic.

I present the new analyses and datasets used to address these concerns and to further explore this particular case of putative adaptation of lexical structure to environmental givens in the following Section “2. Data and methods,” evaluate the results in Section “3. Evaluation,” and conclude with thoughts on what they might mean for the ideas of “efficient communication,” “communicative need,” and adaptability of human languages to their social, ecological, and environmental niches in Section “4. Conclusion: Lexical categories and “efficient communication.” While the results are open to various interpretations, especially when combined with an ethnographic perspective on the societies of the Central Andes, they invite and indeed facilitate a more nuanced view of these notions. This view emphasizes the freedom of linguistic agents to utilize category systems that may or may not conform to these presumed universal principles, barring overly strongly deterministic, mechanistic perspectives on the evolution of category systems.

## 2. Data and methods

### 2.1. Rationale

I will first assess the validity of the results of Urban (2012) by looking at the question of environmental impacts on “cloud”/“fog”- colexification on the basis of a different, non-overlapping dataset, that of the IDS (Key and Comrie, 2021). In order to gain a more fine-grained qualitative and quantitative perspective, however, I will also zoom in on one particular region of the world: the Central Andes. This region corresponds, as the name suggests, to the central part of the Andes mountains of South America, the largest mountain chain in the world. Significant parts of the Central Andes, including the large *altiplano* of Bolivia, are permanently inhabited above 4,000 masl (meters above sea level), making the area eminently suited to investigate the topic. Through a succession of vertically stacked ecozones on the different altitudinal tiers of the mountain chains, there is high ecological and climatic diversity before the mountains finally give way to the Pacific Ocean to the west and the western margins of greater Amazonia to the east. The Central Andes are home to several language families; particular mention deserves the Quechuan family, which has a significant presence throughout the Central Andes, and which, importantly, is represented both on the harsh *altiplano* of Bolivia as well as in the forested lowlands of Ecuador and Peru. Conversely, the Arawakan language family, which clearly has its center of gravity in Amazonia, is represented with the Campa or “Pre-Andine” branch as well as the Yanesha’ language at intermediate altitudes (“intermediate” amounts to a daunting ∼2,500 masl in the Andes). The fact that two well-documented language families spread out across different ecozones and elevations presents the opportunity to trace possible adaptation effects that their lexicon may have undergone (refer also to Urban, 2021 for such effects in Quechuan in a very different context). Such intra-family perspectives are an important complementary piece of evidence to cross-language analyses (e.g., in Everett et al., 2015; Urban and Moran, 2020).

### 2.2. Data

The first global dataset I analyze here comes from the Intercontinental Dictionary Series (IDS, Key and Comrie, 2021). In the latest release, Version 4.2, the IDS provides lexical data for 334 languages and language varieties. Coverage is global but unevenly so. It is very dense for Europe, the Caucasus, and Southeast Asia, good for South America, and poor for North America, Eurasia, and the Indo-Pacific, including Australia and Papua New Guinea. Data have been provided directly by fieldworkers, or in some cases, extracted from published sources by IDS collaborators, with a predefined semantic grid based on that of Buck (1949). It covers a total of 1,310 concepts from different semantic domains (not all cells for concepts are filled for all datasets). One possible danger with the IDS dataset is that, confronted with the task to translate concepts expressed in English into their language of expertise, collaborators might have selected to fill cells with low-salience referents for which there is no real conventionalized lexical expression with a semantically neighboring lexical item (e.g., the word for “cloud” in a language that lags a commonly used equivalent to English *fog*). For ease of analysis, in practice I have treated the presence of one colexifying term as sufficient for coding the language as colexifying, in spite of possible additional terms for either “cloud” and “fog” specifically (I will explore to what extent taking into account non-colexifying synonymy would change the picture in the intra-family analysis reported in Section “2.4. Intra-family analysis”). Colexification behavior was inferred automatically by checking if the number of distinct forms per language corresponding to the IDS concepts “cloud” and “fog” was smaller than the number of total rows in the dataset corresponding to them, which, in accordance with the above operationalization, means that at least one colexifying term is present.

The South American dataset was assembled specifically for this study. It includes data from 78 languages of the Central Andes and adjacent parts, corresponding to the Ecuadorian, Peruvian, and Bolivian parts of the Andes. Coverage is fairly complete, i.e., most (but, due to availability restrictions, not all) languages for which lexical sources (dictionaries or extensive wordlists) are available are included. These sources were matched to Glottolog languoids (Hammarström et al., 2016). There is one issue concerning Quechuan, the largest language family of the Central Andes: In some cases, Glottolog assigns a single Quechua dictionary to more than one variety. Here, each source has been assigned to one, and only one, variety, meaning that one or more of the two varieties were omitted and the source assigned to the variety to which it was deemed to correspond most closely. For instance, Cusihuamán (1976)’s dictionary was treated as a source of Cuzco Quechua and not of Northern Bolivian Quechua (which indeed are very closely related to one another). In dictionaries, there may be more than one word given as the equivalent to either “cloud” or “fog,” and these may or may not be morphologically complex. Languages were coded as having identical terms for “cloud” and “fog” (i.e., as colexifying the concepts) if they feature at least one word that covers both “cloud” and “fog” in the Spanish target language of most dictionaries, corresponding to the translational equivalents *nube*, *niebla*, and *neblina*. If entries for both are given, the term translated to Spanish as *niebla* was given preference over *neblina*, which is more specialized semantically and usually denotes a fine ground fog. For instance, Yanesha is coded as having identical terms—both *nube* and *niebla* are translated as *os*, in spite of the fact that a term translated as “neblina,” *osarets*, is present as well (Duff-Tripp, 1998). This term, in fact, is likely a morphologically complex item headed by *os*. This pattern is typical cross-linguistically (Urban, 2011): Where there is a derivational relationship between items expressing the two meanings, it is usually the one for “fog” that is based on that for “cloud” (which may, as here, colexify “fog”) rather than the other way around, i.e., terms for “cloud” that would translate literally as “high fog” or “sky fog” seem to be much rarer or perhaps even non-existing. Such terms beg the question of how they should be treated analytically—are we dealing with something that is conceptually (and perhaps cognitively) akin to colexification since both concepts are associated lexically? Or are we dealing rather with a case of differentiation, shown by the fact that different (though morphologically related) forms are associated with the different concepts? Here, I evade these questions by reducing the relevant distinctions to a simple and unambiguous distinction between colexification on the one hand and distinct terms on the other hand.

Elevation data for both datasets were retrieved from the 2022 version of the ETOPO Global Relief Model (NOAA National Centers for Environmental Information, 2022) with a 30-arc resolution. The value retrieved for Dutch was negative (which is not implausible given that, indeed, parts of the Netherlands lie below sea level) and was manually set to 1 masl *post hoc* for computational ease.

**FIGURE 1:**
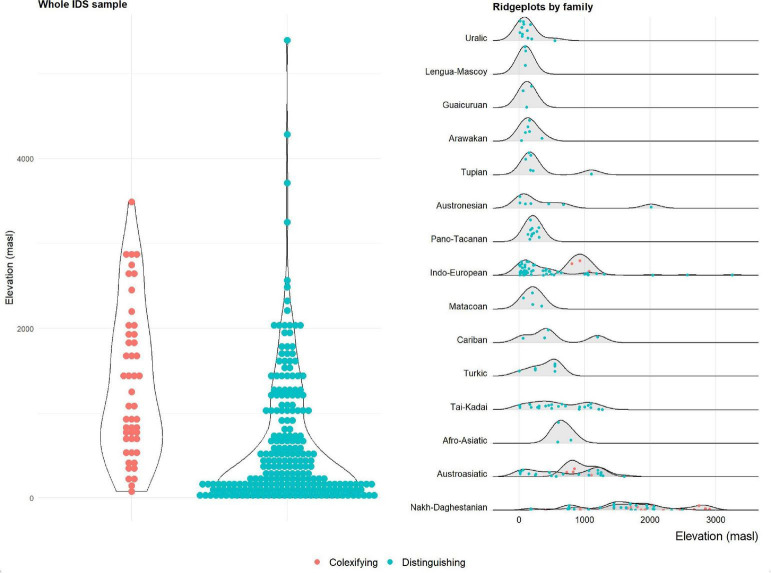
Colexification and non-colexification of “cloud” and “fog” in the IDS dataset, depending on elevation.

**FIGURE 2:**
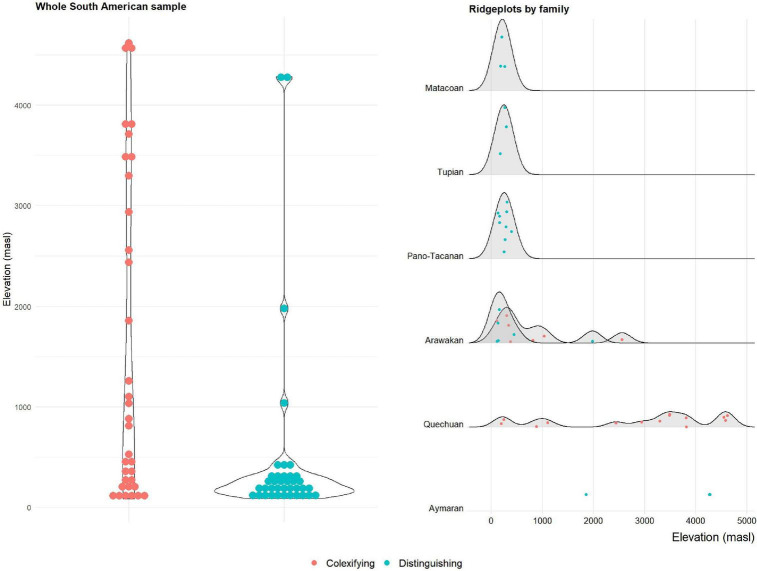
Colexification and non-colexification of “cloud” and “fog” in languages of the Central Andes, depending on elevation.

The panels in Figures 1, 2 show the distribution of colexifying and distinguishing languages depending on elevation (left panels).

The picture obtained from both samples is strikingly consistent, also with the Urban (2012) study. Generally, the mean elevation of colexifying languages is higher than that of non-colexifying languages, consistent with the assumption that elevation has an influence in triggering languages to colexify “cloud” and “fog.” As is evident from the plots, however, the distributions of languages within both groups also differ markedly from one another. Colexifying languages, with few exceptions, center at low elevations in both datasets. In the Central Andean sample, this corresponds to languages of the lowlands to the east, and to a lesser extent west, of the Andes. Languages with distinct terms for “cloud” and “fog,” on the other hand, are less constrained and occur both at the lowest and highest elevations. This is also consistent with the results from Urban (2012), and in contradistinction to the findings of Regier et al. (2016), i.e., there are fewer restrictions on the distribution of colexification but less variance among distinguishing languages, which tend more strongly to cluster at lower elevations.

### 2.3. Cross-language assessment

To assess the role of elevation on the behavior of languages in the sample more formally, I employed two complementary techniques.

First, I resorted to Bayesian mixed-effects logistic regression (Bürkner et al., 2020) for the IDS dataset. Elevation was included after logarithmic transformation due to skew as a fixed effect and language family as a random effect. I placed a conservative, weakly informative prior of SD = 2 on the fixed effect and otherwise used default priors. I ran the models in four chains, with 16,000 iterations each. A total of 8,000 of these were used for warm-up. I, furthermore, increased the drift parameter delta from the default to 0.999 and the maximum tree depth to 20. With these specifications, R̂ values of 1 for each parameter were obtained, and effective sample size estimates and a visual inspection of the chains indicated that the model converged. Comparisons of plots of observed data indicated a good fit of the model to the data. The main effect, altitude (logarithmically transformed), decreased the log odds of observing distinguishing languages by -0.92, with a 95% credible interval of [-1.57, -0.38]. The estimated Bayes factor in favor of the model including elevation as a predictor over a simpler one, which only includes the random effect structure, is 128.49077, providing decisive evidence for the relevance of elevation in shaping the observed distributions.

Applying the same statistical technique to the South American dataset is somewhat problematic because of the many isolates and language families only represented once in the sample (16 out of a total of 28 represented genealogical groups), i.e., levels of the random effect with only one observation; I have, therefore, binned such languages into a pseudo-group, an approach that is methodologically somewhat problematic in spite of being widely applied in more traditional approaches to language sampling (see Miestamo et al., 2016, for a recent instantiation), and then created a model with the same specifications as for the global IDS sample. Here, elevation, again logarithmically transformed, decreased the log odds of observing distinguishing languages by -0.64. Thus, the effect is of a similar magnitude as in the global IDS analysis, though here the 95% credible interval is [-1.63, 0.26] and thus includes zero; in addition, the Bayes Factor estimate of 0.85641 does not provide support for elevation as a relevant factor.

When interpreting this result, one must bear in mind the treatment of isolates and other language families represented only once as one pseudo-group. A further reason for caution is that with a random effects structure that does not collapse isolates and singleton languages to one pseudo-group, the effect becomes still weaker and less credible. Therefore, I have carried out a complementary analysis based on resampling and randomization that avoids this issue (refer to, e.g., Janssen et al., 2007). To this end, 10,000 samples were drawn from the full Andean dataset so that each language family that is represented by more than one language now is only represented by one randomly chosen representative (those only represented once are always included). Then, the variable of interest, i.e., whether or not a colexifying term is present or not, was shuffled for each dataset so that any non-random effect of elevation should disappear. Mean elevations were extracted for both randomized and non-randomized datasets, and the difference was calculated for each of the 10,000 samples.

**FIGURE 3:**
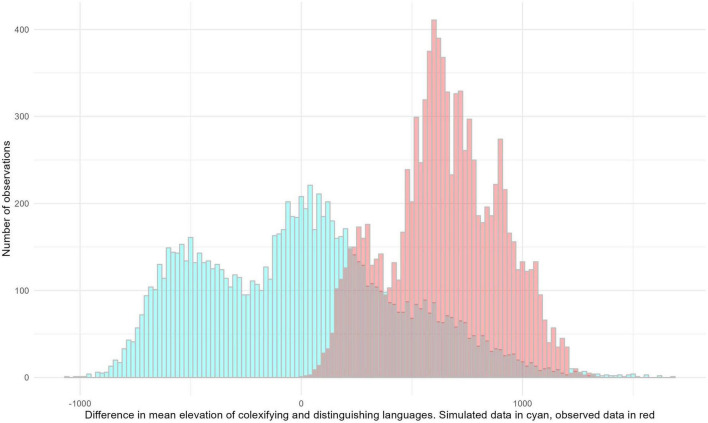
Distribution of the difference between observed elevation means for colexifying and distinguishing languages in 10,000 samples drawn from the full Andean dataset and corresponding simulated means after randomization.

Figure 3 shows the resulting distributions.

Evidently, the distributions overlap, but that for the actually observed values is shifted to the right; that is, mean elevation tended to be higher for the actually observed values, and highly significantly so (Student’s *t*-test 125.6, *p* < 0.00001). The results from Bayesian mixed-effects logistic regression, which suggested that elevation is a significant predictor for “cloud”/“fog”-colexification, are thus robust to this alternative assessment.

### 2.4. Intra-family analysis

The panels in Figures 1, 2 (right side) show an additional aspect of the datasets’ structure, however: there are language families, even language families represented by many languages in the datasets, that are strikingly consistent and only feature languages that are colexifying or distinguishing. In the IDS dataset, the language families that behave this way are exclusively distinguishing (and, consistent with this, have a center of gravity in low-elevation regions). The South American dataset, however, shows that families can also be consistently colexifying. In the Central Andean data, nearly all colexifying languages at high elevations belong to the Quechuan family, whereas the two high-elevation languages with consistently distinct terms are both Aymaran. The results of the analyses in Section “2.3. Cross-language assessment” are robust to this as they ensure that family-specific signals are accounted for. However, the observation suggests that elevation may only be one factor that is at play and that, instead, there may also be lineage-specific preferences, possibly inherited from a common ancestor.

As mentioned earlier, Quechuan is the Native American language family with the largest geographical spread. It ranges from Southern Colombia into northern Chile and Argentina latitudinally and, e.g., from the Pacific-facing Andean environments of Lambayeque in Northern Peru to the western margins of Amazonia in Ecuador and Peru, where Quechuan varieties are spoken in densely forested, hot lowland environments. In other words, the family’s range spans across a set of highly diverse environments that range from low to formidably high elevations.

The Quechuan homeland is disputed, and an earlier theory according to which it lay on the Pacific coast (Torero, 2002) is now increasingly abandoned in favor of a highland origin somewhere in Central Peru (Cerrón-Palomino, 2010; Urban, 2021). The Quechuan spread from that homeland would have been a protracted process, and the farthest peripheries in the north and south would only have been reached in late prehispanic or even historical times. In particular, it is clear that much of the spread into the eastern lowlands is a recent colonial affair that was triggered by missionary action and forced movement of indigenous people (e.g., Zariquiey Biondi, 2004).

Across the family, there are two relevant etyma, *puyu* (seen earlier in the poem) and *pukutay*. Both typically appear in dictionary sources as the translational equivalent of both “cloud” and “fog;” *pukutay*, in addition, often has a verbal reading “to cloud over” (e.g., Yauyos Quechua, Shimelman, 2014) or “to cover with cloud or fog” (e.g., Jauja Wanca Quechua, Cerrón-Palomino, 1976). Emlen (2017), the most recent and most extensive source of proto-Quechua reconstructions, does not reconstruct either term to the proto-Quechua level based on the author’s strict criteria. However, given the wide distribution of both etyma across the family, it is a real possibility that both were present in proto-Quechua already and that they, evidence to the contrary absent, most likely possessed the characteristic colexifying semantic structure.

Under the reasoning outlined in Section “1. Introduction” and according to “efficiency” principles such as those invoked by Regier et al. (2016), this is what would be expected from a proto-language adapted to highland environments. A further expectation in this line of thinking is that Quechuan varieties that reached the lowlands might have gotten under pressure to innovate a distinction. However, in fact, all Quechuan varieties studied are coded as being of the colexifying kind, regardless of the environment and elevation they are spoken in.^1^ To investigate this further, I have looked once more at sources for lowland varieties. As mentioned in Section “2.2. Data,” in the coding scheme for this study, the presence of one colexifying term was sufficient for the language as a whole to be assigned to the colexifying kind. However, there may be additional terms for either “cloud,” “fog,” or both that may represent exactly the sought-after evidence for incipient adaptation to different environments. However, such evidence is largely absent. The only notable innovation among lowland Quechua varieties is that Southern Pastaza Quechua features a separate term for “yellow or red colored clouds” (Tödter et al., 2002), *tsankara*, which is of unclear etymology. Quechuan lexical structure, thus, seems to be highly consistent regardless of elevation.

What is equally striking is that members of the second major highland family of the Central Andes, Aymaran, show exactly the *opposite* patterns. Aymaran is usually assumed to originate in the same or adjacent region as the Quechuan lineage (e.g., Adelaar, 2012); today, it shares the same general highland environment; and some varieties are spoken in overlapping areas in the same environment by bilinguals. However, the consistency with which Aymaran is a distinguishing family is as rigorous as that with which Quechuan is colexifying.

In addition to looking at the question from the perspective of the highland-based families Quechuan and Aymaran, one can also take the point of view of a language family that is clearly lowland-centered, but that has representatives in the immediate vicinity of the Andes at elevations that are already higher than that of the Amazon basin: Arawakan. I have applied generalized linear regression to the Arawakan data in the sample, which features both colexifying and distinguishing languages. However, there was no support for intra-family effects of elevation here either (logit difference + 0.68, SE = 0.64, *z* = 1.11, *p* > 0.05). In addition, the results from the global study, which does suggest a general effect of elevation on language’s behavior, are put into perspective by the observation that there seem to be lineage-specific preferences (see Dunn et al., 2011, for this in a different context) that are operative within (some) families at least at time depths of families like Quechuan and Aymaran (which is generally thought to revolve around two millennia).

## 3. Evaluation

Evaluating the results, we have found support for an impact of elevation on the lexical treatment of “cloud” and “fog,” but crucially, at local levels in relevant environments, such as the Central Andes, this impact may be more weakly distinguishable.

An equally important result is the different distribution of languages in the two groups: Colexifying languages tend to occur at various elevations, whereas distinguishing languages are more constrained to low elevations. In the logic of efficiency in communication that linguists are by now used to as an interpretative framework, for “cloud” and “fog,” we would have to surmise that there is some sort of “incentive” in low-lying environments to distinguish the two, but more freedom to do or not do so otherwise. Colexification might be expected to occur especially in regions such as the New Guinea highlands where perceptual boundaries would be blurred to the extent that the distinction between “cloud” and “fog” made in languages like English loses its meaningfulness. However, in fact, colexifying languages are found at a wide range of elevations. This is in contradistinction to Regier et al.’s (2016) findings, in which the distinguishing languages were less tightly constrained to certain climatic conditions. I have suggested that the case of “cloud” and “fog” may be different from that of “ice” and “snow” studied by Regier et al. (2016) because certain environments render the distinction between the two minimal or non-existent on perceptual grounds, and this may be one part of a more complex and nuanced answer to the question of the conditions under which speakers of languages choose to make or not make distinctions that become reflected in their languages’ category systems. Accounting for such differences in distributions may, in the long run, be more interesting and revealing than assessing the main effect of some environmental variable.

Another major finding is that there are strikingly consistent colexification profiles in language families, regardless of the environment they are spoken in, that retain that consistency, at least at relatively shallow time depths, against larger cross-linguistic trends. The answer to why that is the case is elusive, but it would have to be part of a more complex account of the dynamics of how language structures evolve as well as the conditions and the limits of these processes.

One possible factor that might play a role in explaining the findings is more generalized predilections, indeed adaptations, in balancing lexical richness and semantic generality. As reviewed in Urban (2012: 208–209, 213–216), fieldworkers working on languages as diverse as Vanimo (Papua New Guinea, Ross, 1980) or the Northwest Caucasian languages of the Caucasus (Rayfield, 2002) have noted that extreme restrictions on permissible syllable and word shapes can lead to a lexicon in which items are highly homonymous or polyfunctional, covering a wide semantic space that may or may not be narrowed down by further modifiers. Quechuan languages, indeed, have been noted to be of this kind. Adelaar and Muysken (2004: 233) comment on the “rather limited number of native roots in many domains of Quechua vocabulary. Quechua roots can have a wide spectrum of semantic applications, leaving the impression of a certain lack of semantic differentiation.” *Puyu* and *pukutay* seem to be perfect illustrations here. One of the strengths of Regier et al.’s (2016) study is that, unlike others, it controls for such preferences analytically by examining many word pairs and can thus rule out any possible influence of such language or family-internal profiles. What I would suggest is that lexical typology, including work on adaptive processes, investigate these in their own right rather than treating them as a confound only. There is very little work in this vein from a systematic comparative perspective (with few exceptions, such as Urban, 2012 and Kibrik, 2012).

## 4. Conclusion: Lexical categories and “efficient communication”

In this final section, I offer some more general reflections on the notions of “efficient communication,” “communicative need,” and their relationship to lexical categories, departing from the results of the present study, in particular that of the Central Andes.

In the Andes, freshwater and rainfall are of paramount importance, and so are clouds and fog. Not only has atmospheric moisture shaped an entire Andean ecosystem, the tropical montane cloud forests (Helmer et al., 2019), but it also is of direct, vital relevance for human subsistence and culture in the communities that support Central Andean languages and in the context in which they evolved. For example, in the highlands, people are able to predict rainfall and hence, harvest on the basis of barely visible high cirrus clouds that form only under El Niño conditions and that dim the Pleiades at night in certain years, forespelling dry conditions and poor harvests (Orlove et al., 2000, 2002); the dread such forecasts bring to communities is documented vividly in Urton (1982). At lower elevations, so-called *lomas* are micro-ecozones in which frequent fog creates enough moisture to locally sustain vegetated areas with the concomitant affordances for humans in an otherwise hyperarid desert environment. In the maritimely oriented coastal societies, < potossis > , a term of unclear but obviously indigenous origin, is a term denoting “thin and transparent white clouds which appear on the Milky Way in clear and moonless nights and which announce abundance of fish” (Rodríguez Suy Suy, 1997: 90).

Given such ethnographic evidence, atmospheric phenomena related to moisture appear to be among “the chief interests of a people,” to take up the phrase from Boas, (1911, p. 26), in many, perhaps all, parts of the Central Andes. Under an interpretation in terms of efficiency principles such as that of Regier et al. (2016), this should entail “the need to communicate precisely and informatively” about them, and that, in turn, should entail a category system that facilitates such communication. But if the standard to measure the effectiveness of such a system are lexical distinctions, then the category system of languages such as those of the Quechuan family fails to support the reasoning.

The idea that languages are adapted for efficiency while also under pressure from the opposing force of clarity of expression as required for successful communication has a long pedigree, clearly expressed in Von der Gabelentz (1901: 181–5), taken up in Prague School phonology (Martinet, 1952), and in the Zipf (1949) approach to human (linguistic) behavior. Some kind of adaptation for communicative efficiency is now argued for by a wealth of studies ranging across different domains of language (e.g., Bentz, 2018; Coupé et al., 2019; Gibson et al., 2019; Levshina and Moran, 2021). The idea of environmental factors triggering adaptive processes, in fact, is part of a larger family of reasoning in which languages are said to be adaptive to biological (e.g., Dediu et al., 2017), cognitive (e.g., Pinker, 2003), and social (e.g., Lupyan and Dale, 2010) environments.

For quite some time, thus, and especially in the most recent past, linguists have become used to the idea that language structures evolve in response to the communicative tasks they need to fulfill relative to biological, cognitive, social, and, according to some, environmental environments.

Lest I be misunderstood, my aim is not to downplay findings that support such ideas (in fact, the statistical evaluation offered here does so to a considerable extent) or to trivialize them. Nor do I wish to perpetuate a stance in which any possibility for adaptive effects is denied *a priori* for theoretical reasons. My plea, however, is that aspects of the evidence such as the Quechuan one, which are not readily accounted for by the main thrust of the argument, not be dismissed lightheartedly. There is a variety of additional assumptions that might be employed to accommodate the observed behavior to the interpretative framework. For instance, it is well possible that the post-1492 expansion of Quechuan to the lowlands may simply be too recent for any adaptive processes to affect the lexicon yet. However, given that we know next to nothing about the time frames that would be required for such putative processes to set in, this would be an unmotivated *ad hoc* assumption. Making such an assumption (perhaps under a confirmation bias) possibly obscures other aspects of the formidably complex tangle of factors that shape the development of languages. The non-adaptiveness of Quechuan lexical structure with regard to the distinction between “cloud” and “fog” may be indicative of these. Like other contemporary research, what they do show rather clearly is that the relationship between language and environment is in no way deterministic: Even if we assume an interpretative framework of communicative efficiency of one sort or another by which, indirectly, environments shape language structure, language users, such as the speakers of Quechuan languages, but also others, are free to develop and maintain structures that, judged from the abstract perspective of efficiency, would seem counterproductive, and communicate with these effortlessly.

In addition, I would also like to draw attention to the ways in which language use, in language- or region-specific ways, can shape category systems that both demonstrate the creativity of speech communities and, at the same time, arguably also the evolution of structures that may be considered environmentally adapted.

On the one hand, these involve apparently unstructured lexical specializations of the “eskimo words for snow” type. These are usually considered trivial—they are language- and environment- specific and are unlikely to be meaningfully amenable to cross-linguistic investigation. It would make little sense, for instance, to compare terms for the desert landscape on which the Southern Paiute live (Sapir, 1912, p. 229, who incidentally, like Boas, holds that these do not reflect the environment *per se*, but rather the “interest of the people” in them) with those of languages where they likely simply lack comparable equivalents.

However, I do believe that there is a way other than the study of colexification patterns in which processes that might be termed adaptive can arise. These are of a less trivial kind in that they pertain not to assorted collections of lexical items in a particular semantic domain, but rather concern underlying organizing principles in environment-related semantic domains. These relate to specific ways in which language users create and maintain them and thus dovetail with the lineage-specific preferences for either colexification or differentiation in the aerosol domain observed in this study.

Here, I am referring to phenomena of two kinds: One are semplates in the sense of Levinson and Burenhult (2009), whose examples, perhaps not coincidentally, are drawn from the domain of topographic reference. Many of these are language-specific schemata whose structure references the environment systematically along axes that often correspond to physical features and are usually overlain by cultural associations. The Tzeltal uphill/downhill distinction is a well-known case of a system of spatial cognition that is conventionalized to a large degree in discourse but ultimately “inspired” by the sloping Tzeltal lands (Brown and Levinson, 1993). Another example is systems of elevation deixis such as those found in Himalayan languages, in which, with distinct cultural overtones, the same elevation contrasts recur across lexical items of different parts of speech, e.g., demonstratives and verbs of motion (Ebert, 1999).

**FIGURE 4:**
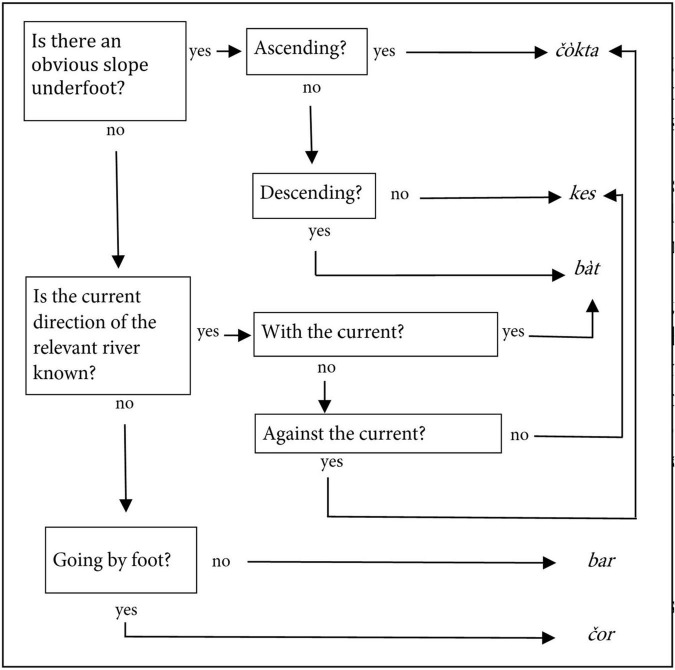
Flowchart for Tuvan verbs of motion in topographic orientation, redrawn from Harrison (2007: 128).

Such stable cross-domain mappings of environmental variables are now also coming into the purview of comparative work, with mixed results (Palmer et al., 2017; Forker, 2019). However, there may also be other ways in which linguistic structures adapt systematically to aspects of the environment, which, like the examples just mentioned, are notably anchored in the overall system of cultural knowledge of the societies that support the relevant languages. Here, I have in mind mappings such as those in at least Southern Peruvian Quechua (but likely present elsewhere as well) of *qhiswa* “temperate valley” and *puna* “high plateau” onto distinct lifestyles on the respective ecological zone (Isbell, 1978). There is a linguistic dimension to this in that *sara* “maize” and *papa* “potato,” which are the quintessential agricultural products of the respective ecological zones of the Andes Mannheim (1998, p. 264), repeat the same underlying classification but without any overt indicator of that, just like a semplate. Another more complex example is topographic orientation in the Tuva language of Siberia, as discussed by Harrison (2007: 127–130) and summarized by him in a flowchart that is redrawn here in Figure 4. Obviously attuned to an environment characterized by a sloping terrain in which rivers flow, this is a clear case of linguistic adaptation to the geophysical environment.

Similar to the case of the Southern Paiute’s landmark terminology, these lexical categories are unlikely to be amenable to large-scale comparative perspectives, and for the same reasons, i.e., the very fact that they are attuned to specific environments and only make sense in these (though refer to Holton, 2011, for a small-scale qualitative comparative study). However, that does not mean that they are not linguistically and cognitively real, nor that they cannot be considered a way in which non-linguistic factors, indeed, shape language structure. There is something else that is remarkable about them: in spite of the heterogeneity of the examples just cited, authors emphasize how the systems of spatial and environmental reference and nomenclature are embedded into broader cultural schemas that integrate them into an organic system of making sense of the world. At the same time, they clearly portray the communities that created such systems as agents shaping linguistic categories actively and creatively as “language builders” (Hàgege, 1993) in response to the environment they find themselves in. They thus bar, just like the consistent preferences for or against colexification in some families against global trends, strongly deterministic views on the processes in which the categories of languages are molded, and, like much recent research on adaptive processes in languages, invite to explore the tangle of factors that shape the structures of languages in all its complexity.

## Data availability statement

The Andean dataset analyzed in this study and R code to reproduce all analyses are available from GitHub at https://github.com/urban-m/cloudfog.

## Author contributions

The author confirms being the sole contributor of this work and has approved it for publication.

## Footnotes

1 A possible exception is North Junín Quechua as documented by Adelaar (1977). In this highland variety, “cloud” is *pugutay* ∼ *pukutay*, and in the Tarma dialect, there is in addition the specialized term *xuĉa* “dark rain cloud.” This, interestingly, reflects proto-Quechua *qucha, a general term for standing bodies of water such as ponds, lakes, and even the sea. *xučxča*- is a verb meaning “to be foggy” in the San Pedro de Cajas dialect, while it means “to smoke (chimneys, etc.)” in the Tarma dialect. A nominal form for “cloud, fog” apart from *xuĉa* is not mentioned. Adelaar (p.c.) emphasizes that his data come from few individual speakers so that idiolectal factors cannot be excluded.
